# Has Covid-19 Weakened Workplace Socialization? Associating Outcomes of Excessive Work Time With the Entrepreneurial Behavior of Employees

**DOI:** 10.1177/00332941231207956

**Published:** 2023-10-24

**Authors:** Sana Mumtaz, Tanzeela Aqif

**Affiliations:** Shannon School of Business, 55964 Cape Breton University, Sydney, NS, Canada; FAST School of Management, 119728 NUCES, Islamabad, Pakistan

**Keywords:** Creative self-efficacy, employee resilience, entrepreneurial behavior, excessive work time, social identity theory, socialization tactics

## Abstract

The Covid outbreak and the resulting work processes have led to excessive work pressure requirements for a large majority of the employees due to continuous requirements for adaptation to changing work processes. However, outcomes of new work dynamics on employees’ behavioral and attitudinal changes have been insufficiently examined during and after the Covid period. Hence, this empirical research has used the social identity theory to conceptualize and test the relationship between excessive work time and the entrepreneurial behavior of employees. Moreover, the mediating role of socialization tactics and creative self-efficacy, as well as the moderating role of resilience are also examined in the above relationship for unveiling the role of crucial intervening and conditional factors in individualized change experiences. Based on the analysis of data collected from managerial-level employees through the use of structural equation modeling, the empirical results have suggested a negative impact of excessive work time on the entrepreneurial behavior of employees. Also, the intervening role of formal socialization and creative self-efficacy were found to be significant in this relationship, thus suggesting the crucial direct as well as indirect relationships. Using the findings, implications as well as future research directions are discussed towards the end of the article.

## Introduction

While organizations have offered newly developed guidelines and policies regarding work standards for various types of jobs, employees’ work hours and time-based requirements have emerged as a varying factor as many employees are bound to spend more than the required time on organizational work activities and goals achievement (Palumbo, 2020). Overall, there was a fine distinction between work and non-work time distinction for employees in most of the sectors before Covid-19, however the work processes and dynamics have drastically changed in the last few years, especially with regard to the allocation of work time for goals achievement (Wang et al., 2020). One of the major changes is that most employees are now required to spend excessive work time for timely achieving their goals because of work-from-home and flexible work arrangements. While excessive work time has been discussed as an area of concern in some literature, which has linked it with weak physical health and negative performance of employees (Di Stefano & Gaudiino, 2019; Wada et al., 2019), there is almost no guidance exists regarding individualized change experiences and behavioral outcomes for employees after spending excessive work time in organizations.

Alongside the above, it is crucial to identify the underlying process or mechanism which leads to positive or negative change experiences in such individuals who dedicate excessive work time to organizations. In this regard, some of the literature has remained confined to negative change experiences of excessive time for healthcare professionals after Covid-19 (e.g. Kabasakal et al., 2021), however it is important to understand how and under what conditions such experiences might uniquely lead to positive changes in some individuals. However, there has been limited focus on the integration of both aspects in a single model. In view of the same, this research has focused on unveiling the complex mechanism which leads to positive or negative changes in employees because of spending excessive work time in organizations after Covid-19.

To conceptualize a model explaining individualized outcomes for employees, the social identity theory has been integrated into the research, which focuses on the essence of intergroup relationships and the essence of social group formation in diverse contexts (Tajfel & Turner, 2004). This theory emphasizes socialization as a basis for the change experiences of individuals (Bornewasser & Bober, 1987) and has been integrated into some literature for highlighting how lack of interactions prohibits individuals from experiencing positive changes over time (e.g. Mumtaz & Nadeem, 2022). Thus, it has been used in this research to suggest that excessive work time is likely to impact the entrepreneurial behavior of employees because of the use of digital communication tools and frequent changes in organizations’ ways of work (Jong, Parker, Wennekersb & 2015). Entrepreneurial behavior refers to the set of characteristics, attitudes, and actions displayed by individuals who exhibit traits like creativity, risk-taking, initiative, innovation, and a proactive approach to problem-solving (Bird & Schjoedt, 2017). While the term “entrepreneurial” is associated with business founders and owners in general (Thompson, 2009), it may be applied to employees within an organization who demonstrate these qualities and behaviors. Before the Covid-19 pandemic, entrepreneurial behavior in employees was gaining recognition as a valuable asset for businesses as many companies encouraged and fostered a culture of innovation, allowing employees to take initiative and contribute their ideas to drive growth and success (Escrig-Tena et al., 2022). However, the pandemic brought about significant changes in the business landscape, such as rapid adaptation to new circumstances and a need for greater adaptability and resilience among employees for change management (Jiatong et al., 2022). In recent times, employees are constantly overwhelmed and stressed because of excessive work time, thus their capacity for creative thinking and problem-solving has diminished. Also, Entrepreneurial behavior relies heavily on creativity and innovative thinking, so when these qualities are compromised, employees may struggle to come up with new ideas and initiatives (Faghri et al., 2021).

Along with the direct impact of excessive work time on the entrepreneurial behavior of employees, it is expected that formal socialization and creative self-efficacy are likely to indirectly impact the above relationship. In this regard, it is suggested that excessive work time is likely to discourage employees from engaging in formal socialization because of fewer interaction opportunities (Easa & Bazzi, 2021), which might also reduce their creative self-efficacy. Entrepreneurial behavior is often fueled by interactions with colleagues, brainstorming sessions, and collaborative efforts. When employees are isolated or have limited opportunities to interact with others, sharing and developing ideas collectively becomes challenging (Jeong & Shin, 2019). Thus, weak socialization may result in reduced collaboration and limited opportunities for idea exchange. Moreover, employees with low creative self-efficacy may hesitate to generate new ideas or believe that their ideas are not valuable or innovative enough (Garaika et al., 2019). As a result, they may refrain from proposing new initiatives or solutions, hindering entrepreneurial behavior driven by innovation and creativity.

The following are the key contributions of this research. First, this research has developed a linkage between excessive work time and the entrepreneurial behavior of employees, hence taking into perspective long-term negative change experiences of excessive work time on the behaviors and attitudes of employees. Second, the research model has discussed the unique role of intervening and conditional variables such as socialization tactics, creative self-efficacy and resilience in the above relationship, thus helping in identifying a process of change in employees over time. Overall, the research model provides a basis for understanding the essential role of socialization in impacting the long-term psychology and mindset of employees and needs to be thoroughly investigated in diverse contexts for theory building in the domains of organizational behavior and social psychology.

## Literature Review and Hypothesis Development

**Figure 1.**
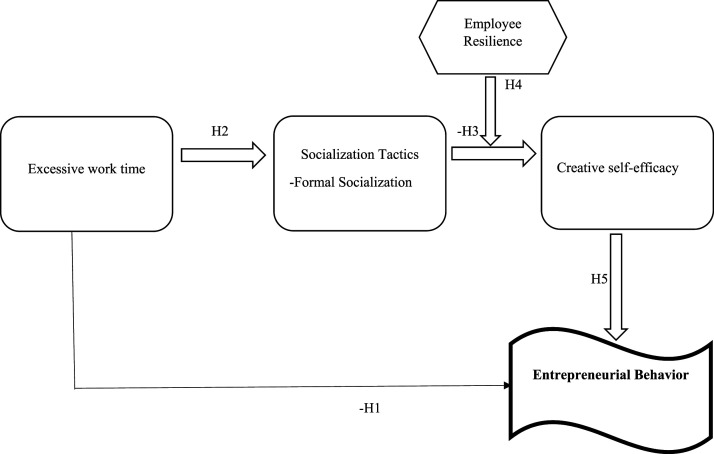
A Process Model linking Excessive Work Time with the Entrepreneurial Behavior of Employees.

This section provides an overview of the literature on key variables as well as connects the social identity theory with the literature for developing thrade model of the research. This model develops a process model which links excessive work time during Covid with the entrepreneurial behavior of employees. The summarized model is presented in Figure 1.

### Relating Excessive Work Time With Entrepreneurial Behavior

In general, a large number of organizations across the globe have identified minimum time requirements for employees for ensuring standardization and objectivity in their processes. While there is flexibility in the work schedule of employees according to the nature of their responsibilities, most organizations require employees to spend 40 hours a week or 8 hours a day on average (Johnson & Lipscomb, 2006). While the above time requirements were widely accepted by employees and became a part of their routine HR processes, the recent pandemic and the consequent work-from-home arrangement have drastically altered the above policies as it has become extremely challenging for organizations to precisely measure the amount of time being spent by employees on their work (Wang et al., 2020). While the recent literature provides little about the work timings of employees after Covid (Diab-Bahman & Al-Enzi, 2020), this is an important area of research as work has become more challenging for most employees after Covid-19 due to organization’s adaptation to new systems and procedures. Consequently, employees are dedicating excessive work time to goals despite working from home. Overall, the negative outcomes of excessive outcomes on the physical and mental health of employees have been elaborated in some literature (e.g. Caruso, 2014; Di Stefano & Gaudiino, 2019; Rosa, 1995), however there is almost no understanding regarding how the recent pandemic and consequent excessive work time from home has changed employees’ mindset and their performance.

In view of the above gaps, this research has used the social identity theory to propose the relationship between excessive work time from home and entrepreneurial behavior. The social identity theory illustrates that intergroup relationships between individuals and strong relationships facilitate them in embracing positive changes in their behavior and attitude over time (Tajfel & Turner, 2004). Moreover, it gives an explicit sense to individuals about their position in various groups, such that a lack of congruence might reduce their confidence and leads to negative social identity changes (Brown, 2000). Thus, using this argument and the literature on organizational behavior, it is suggested that during Covid, most employees were bound to spend more than the required hours for work even while working from home, which has reduced their propensity to interact within their social circle (Easa & Bazzi, 2021). Thus, excessive work from home during and after Covid has weakened the entrepreneurial behavior of employees, which refers to an ability to come up with novel ideas and solutions for continuous improvements (Moore, 1986). It is argued that employees’ focus on existing work processes and adjustment to a new system is likely to hinder their creative capabilities, thus might lead to a negative impact on their entrepreneurial behavior. Based on the above discussion, Hypothesis 1 is developed:

Hypothesis 1 Excessive work time has adversely impacted the entrepreneurial behavior of employees during and after Covid-19.

### Relating Excessive Work Time With Formal Socialization Tactics

The social identity theory illustrates the essential role of interactions and frequent conversation between a member of diverse groups, and how such discussions help individuals deeply understand the expectations and behaviors of other people (Bornewasser & Bober, 1987).). Such discussions are regarded as essential in the organizational context since employees go beyond their work-based interactions and occasionally share things from their personal lives with other employees, eventually improving their social relationships (Mumtaz & Nadeem, 2022). While the essence of socialization has been reflected in a sufficient body of literature, there has been a very weak understanding of how the recent pandemic has changed the intensity of socialization between organizational members.

Keeping in view the above, it is suggested that the Covid period has especially impacted the socialization pattern among organizational employees as new ways of work and excessive pressure regarding conformity with changing dynamics have led to formal socialization between employees. Thus, most of the employees remained confined to their individual goals and avoided extra discussions as a result of spending excessive work hours from home. Moreover, the use of formal socialization mechanisms and online mediums has especially confined employees to work-based discussions. Based on the above discussion, Hypothesis 2 is developed:

Hypothesis 2 Excessive work time has led to the development of formal socialization among employees during and after Covid-19.

### The Moderating Role of Employee Resilience

The essence of socialization has been highlighted in a large body of literature (e.g. Cohen & Veled-Hecht, 2010; Mumtaz & Nadeem, 2022), which illustrates how frequent interaction facilitates employees in easily overcome routine conflicts and misunderstandings and helps them in developing long-term trust with each other. Although a formal socialization mechanism also offers an opportunity to share their perspectives and feedback, however the use of a confined and pre-decided discussion plan prohibits employees from discussing novel ideas. Thus, it is likely to negatively impact the creative self-efficacy of employees. Creative self-efficacy refers to individuals’ faith regarding their creative capabilities and self-belief that they can come up with novel solutions and plans in case of ambiguous challenges (Tierney & Farmer, 2002). Using the social identity theory, and the essence of informal interactions between colleagues in their learning process, it is suggested that formal socialization tactics are likely to weaken the creative self-efficacy of employees.

It is crucial to understand that personality-based differences play a vital role in differentiating the socialization needs of employees. Further, the social identity theory offers explicit guidance regarding the role of individuals’ thinking processes and values in social group formulation (Hogg, 2001). Thus, it is suggested that employee resilience is likely to conditionally impact the above relationship. Employee resilience reflects individuals’ capacity to adjust well to changing dynamics and their internal passion toward a goal (Kuntz et al., 2017), which enables them in viewing opportunities under new circumstances, rather than threats. Hence, it is suggested that employee resilience is likely to uniquely shape the intensity of the above relationship as its presence would help employees in improving their creative self-efficacy despite the presence of a formal socialization mechanism. Overall, employees are likely to enhance their skills and try to use new tools of learning in this situation, which would weaken the relationship between formal socialization tactics and creative self-efficacy. Based on the above discussion, Hypothesis 3 and 4 are developed:

Hypothesis 3 Formal socialization between employees has adversely impacted the creative self-efficacy of employees during and after Covid-19.

Hypothesis 4 Employee resilience moderates the negative relationship between formal socialization mechanism and creative self-efficacy, such that their negative relationship gets weakened for employees having high resilience as compared to low resilience.

### Relating Creative Self-Efficacy With the Entrepreneurial Behavior

The previous section provided brief arguments regarding how employees’ excessive work time during Covid and less interaction weakens their creative behavior. However, the presence of resilience facilitates individuals in overcoming this barrier and in improving their creative self-efficacy. While most of the existing social identity literature has focused on individuals’ relationships with new groups and positive change experiences of such interactions (e.g. Mumtaz & Nadeem, 2022), it is important to understand how fewer interactions lead to positive changes in individuals occasionally. Focusing on the above, it is suggested that the use of creative self-efficacy is likely to boost the self-confidence of employees and enable them to improve their learning and exposure. It is argued that fewer socialization opportunities during Covid and the creative self-efficacy of employees will motivate many individuals to enhance their learning and to come up with novel ideas and new opportunities. Thus, creative self-efficacy is likely to improve the entrepreneurial behavior of employees and reduce their dependencies on other people (Newman et al., 2018). Hence, such employees are likely to grow more in their careers and think about their ventures as a result of excessive work time and learning exposure. Based on the above discussion, Hypothesis 5 is developed:

Hypothesis 5 Creative self-efficacy has improved the entrepreneurial behavior of employees.

## Methodology

### Sample and Procedures

The main objective of this research was to understand whether and how excessive work time during Covid-19 has led to changes in the entrepreneurial behavior of employees. Based on the objectives of the study, data were collected from managerial-level employees working in the government and private sectors of Pakistan. The purposive sampling technique was used for collecting data from respondents. This study aimed to collect data from young, mid-age, and senior employees to holistically understand the change experiences of employees at various levels. Data were collected through Google Forms and respondents were assured about the confidentiality of their valuable responses. Overall, the purposive sampling technique was used to initially send questionnaires to around 500 targeted respondents through online mediums such as LinkedIn and email and requested them to fill out a Google form. The questionnaires were directly sent to the employees of both public and private sector organizations. Overall, 500 questionnaires were distributed and the number of obtained responses was 279, hence the response rate was around 62%.

The analysis of the demographics indicated that 68.2% of males and 31.2% of females participated in this study. Demographic results further showed that 37.7% of participants were working at junior-level positions in their respective organizations, while 40.2% were working at middle-level managerial positions. Around 22.1% of them were working in senior-level positions. The results also indicated that 78% of participants never thought of having their businesses while 22% aim to start their own businesses soon.

## Measures

### Excessive Work Time

Excessive work time was measured using a four-items scale developed by Peetz and colleagues (2004). All the questions were measured on a five-point scale ranging from 1 = Strongly disagree to 5 = Strongly agree. Following is a sample item: ‘*If you take time off or get sick, your work just builds up while you’re away’*. The Cronbach’s alpha was .89.

### Formal Socialization Mechanism

Formal socialization mechanism was measured using a five-items scale developed by Jones (1986). All the questions were measured on a five-point scale ranging from 1 = Strongly disagree to 5 = Strongly agree. Following is a sample item: ‘*I have been through a set of training experiences which are specifically designed to give us knowledge of job-related skills’*. The Cronbach’s alpha was .73

### Employee Resilience

Employee resilience was measured using a nine-items scale developed by Naswall and colleagues (2019). All the questions were measured on a five-point scale ranging from 1 = Strongly disagree to 5 = Strongly agree. Following is a sample item: *‘I can work on task that allow for my evaluation’*. The Cronbach’s alpha was .81.

### Creative Self-Efficacy

Creative self-efficacy was measured using a five-items scale. All the questions were measured on a five-point scale ranging from 1 = Strongly disagree to 5 = Strongly agree. Following is a sample item: ‘*I am willing to master knowledge I need for creative tasks’*. The Cronbach’s alpha was .83.

### Entrepreneurial Behavior

Entrepreneurial behavior was measured using a 22-items scale developed by Lumpkin & Dess (1996). All the questions were measured on a five-point scale ranging from 1 = Strongly disagree to 5 = Strongly agree. Following is a sample item: ‘*I can assume any kind of risk if I believe in the success of the task’*. The Cronbach’s alpha was .73.

## Results

To compute statistical results, SPSS and Smart PLS 3 software have been used in this study. Initially, the correlation analysis has been performed in the SPSS software to examine the relationship among variables. Moreover, the demographic characteristics of sample were computed. Afterward, structural equation modeling (SEM) was conducted and measurement model assessment was performed to validate the model. For model testing, the bootstrapping technique has been used and ‘*p*-values’ (with 95% significance level) were interpreted for accepting or rejecting hypothesis.

### Correlation Analysis

Table 1 presents the details of descriptive and correlation analysis for all the variables included in the study. The results indicated a moderate positive correlation between excessive work time and formal socialization (r = .58, ρ < .001). Further, a moderate correlation was found between excessive work time and creative self-efficacy (r = −.63, ρ < .001). The results indicated a strong negative correlation between excessive work time and the entrepreneurial behavior of employees (r = −.73, ρ < .001).

**Table 1.** Descriptive and Correlation Analysis.

	Mean	SD	1	2	3	4	5
1. Excessive work time	3.02	.63	-				
2. Formal socialization	3.01	.59	.58**	-			
3. Creative self-efficacy	2.96	.61	−.63**	−.68**	-		
4. Employee resilience	3.21	.82	.50*	.53**	−.61**	-	
5. Entrepreneurial behavior	3.02	.73	−.50*	−.59*	.63	−.73**	-

### Confirmatory Factor Analysis

The discriminant and convergent validity of constructs was examined through the confirmatory factor analysis. The analysis has been performed through PLS principal component analysis (PCA). Results indicated that factor loadings for all items were above .7 on their respective variables which established the validity of constructs. Similarly, the values of composite reliability were more than .8 in line with the guidance of the literature. The values of Average Variance Extracted were also found to be above the threshold level (<.5). Table 2 includes items of all constructs included in the analysis, factor loadings, CR, and AVE along with factor loadings of items.

**Table 2.** Confirmatory Factor Analysis.

First-Order Constructs	Items	Factor Loadings	Composite Reliability (>.7)	AVE (>.5)
Excessive work time	EWT1	.93	.88	.74
	EWT2	.88		
	EWT3	.76		
	EWT4	.73		
	EWT5	.84		
Formal socialization	FS1	.80	.89	.77
	FS2	.82		
	FS3	.75		
	FS4	.92		
	FS5	.85		
Creative self-efficacy	CSE1	.70	.89	.64
	CSE2	.74		
	CSE3	.60		
	CSE4	.83		
	CSE5	.81		
Employee resilience	ER1	.74	.90	.74
	ER2	.86		
	ER3	.80		
	ER4	.82		
	ER5	.77		
	ER6	.74		
	ER7	.87		
	ER8	.81		
	ER9	.83		
Entrepreneurial behavior	EB1	.86	.91	.82
	EB2	.78		
	EB3	.81		
	EB4	.84		
	EB5	.72		
	EB6	.71		
	EB7	.79		
	EB8	.72		

### Regression Analysis

The model was tested using the structural equation modeling technique in the Smart PLS software. The values of standardized coefficients were interpreted and a significance level of 5% was selected for accepting the results in light of the guidance of the literature. It was proposed in Hypothesis 1 that excessive work time leads to a negative impact on the entrepreneurial behavior of employees. In line with the proposition, the results indicated a negative impact of excessive work time on the entrepreneurial behavior of employees (β = −1.023, *p* < .001). Therefore, Hypothesis 1 was accepted. Hypothesis 2 proposed a positive impact of excessive work time on formal socialization tactics. The statistical results were aligned with the proposition (β = .794, *p* < .001). Hence, Hypothesis 2 was accepted. In Hypothesis 3, a negative impact of formal socialization was predicted on the creative self-efficacy of employees. Contrary to expectations, the results indicated a positive impact of formal socialization tactics on the creative self-efficacy of employees (β = .741, *p* < .001). In line with the results, Hypothesis 3 was not accepted. Hypothesis 4 proposed a moderating role of resilience in the relationship between formal socialization and creative self-efficacy. The interaction effect was found to be insignificant (β = −.069, ns), therefore Hypothesis 4 was not accepted. Finally, a positive impact of creative self-efficacy was predicted on the entrepreneurial behavior of employees in Hypothesis 5. The results indicated a positive impact of creative self-efficacy on the entrepreneurial behavior of employees (β = .818, *p* < .001), thus Hypothesis 5 was accepted.

Along with direct effects, indirect effects were also analyzed for understanding the crucial role of intervening variables in the model. The statistical results indicated significant negative results regarding the role of formal socialization in the relationship between excessive work time and creative self-efficacy (β = −.827, *p* < .001), as well as creative self-efficacy was found to have a significant negative effect on the relationship between formal socialization and entrepreneurial behavior (β = −.596, *p* < .001). These results suggested the presence of direct as well as indirect negative relationships between excessive work time and entrepreneurial behavior. The results of path analysis are presented in Table 3.

**Table 3.** Results of Path Analysis.

Structural Path	Estimate	*p*-Value
Direct effects		
Excessive work time → entrepreneurial behavior	−1.023	.001
Excessive work time → formal socialization tactics	.794	.000
Formal socialization → creative self-efficacy	.741	.000
Formal socialization*Resilience → creative self-efficacy	−.069	.105
Creative self-efficacy → entrepreneurial behavior	.818	.000
Indirect effects		
Excessive work time → formal socialization → creative self-efficacy	−.827	.000
Formal socialization → creative self-efficacy → entrepreneurial behavior	−.596	.000

## Discussion

This research has developed and tested a unique model linking excessive work time with the entrepreneurial behavior of employees post-Covid period using the social identity theory. Based on the findings of this study, theoretical implications of this research along with future research directions are discussed in this section.

This research has extended the boundries of the existing knowledge by focusing on the outcomes of excessive work time on the behavior of employees after the Covid period. The findings of the Hypothesis 1 establishes a negative linkage between excessive work time and the entrepreneurial behavior of employees, and adds value in the literature by suggesting how time-bound requirements cannot be necessarily associated with work goals and accomplishements of employees, but leads to adverse impact on the attitudinal and behavioral responses of employees. While the concept of excessive work time has been discussed in some literature (e.g. Caruso, 2014; Okazaki et al., 2019), this is one of the first few studies that has developed a process model for unveiling outcomes in a sequential way. Future researchers are suggested to expand their understanding regarding the outcomes of excessive work time on various other work and non-work outcomes, such as researchers may examine the outcomes on the career progression of these individuals using the longitudinal research designs. Also, the integration of the above literature with other domains such as health sciences may help in understanding the long-term prospects of excessive work time on the physical and mental health of employees.

Moreover, the findings of Hypothesis 2 confirm the positive impact of excessive work time on the development of formal socialization between employees. These findings add value to the literature by suggesting that when employees are indulged in work all the time, they are less likely to get an opportunity to engage in informal socialization and confine themselves to work-related affairs only. Moreover, these findings align with the notion of the social identity theory which focuses on the idea of ties development through frequent socialization and interactions. Contrary to the expectations, the findings of Hypothesis 3 suggested a positive impact of the formal socialization mechanism on the creative self-efficacy of employees. The reason behind these results may be interpreted in light of the identity perspective and personality-based differences between individuals. There is a likelihood that the formal socialization mechanism might have facilitated employees in thoroughly understanding work-related goals and helped them in becoming more independent in terms of decision-making and resolving problems using the distinct approach. Hence, they experienced a positive change in their creative self-efficacy over time. In view of these results, researchers are recommended to focus on examining and testing the positive aspects of formal socialization on the social psychology and attitude of individuals. Also, future researchers may incorporate the conditional role of personality-based factors in this model for thoroughly understanding individualized differences in-depth. In Hypothesis 4, the moderating role of employee resilience was proposed in the relationship between formal socialization and creative self-efficacy, however statistical results did not provide support for this relationship. The insignificant role of employee resilience may be interpreted in light of the complex and multifaceted nature of the concept of resilience which is likely to be developed when individuals experience uncertainty. However, employees might did not experience any personalized changes in their perceptions during and after the Covid period. In order to understand the role of employee resilience, future researchers are suggested to conduct in-depth interviews with employees regarding their individualized change experiences.

Based on the Hypothesis 5, the findings of the research suggested a positive impact of creative self-efficacy on the entrepreneurial behavior of employees. This finding adds value to the organizational behavior literature by highlighting how individuals can develop a unique mindset toward novel practices and become more creative through self-independence with time. Also, such perceptions make employees think about new business solutions on a wider scale. While the findings of this research serve as a basic milestone in understanding the changes in employees’ mindset and behavior due to excessive work time, it would be interesting for future researchers to explore the outcomes of these changes on employees’ propensity of initiating new business ventures in the future. In this regard, the use of qualitative research designs might provide comprehensive guidance to researchers for understanding whether and how employees shifted towards their ventures in the future.

Overall, the findings of this research offered an understanding of direct relationships between various variables as well as unveiled the essential role of intervening factors in indirectly impacting the change experiences and thought processes of employees who spend excessive time on work, particularly after the Covid period. This model adds value to the organizational behavior and social identity literature by suggesting a process that links various behaviors with the cognitions and social psychology of employees. To expand theory in this domain, future researchers are encouraged to integrate diverse theoretical approaches and models for unveiling other essential factors and conditions in this change process. Moreover, this research was conducted in the Asian context where employees are more proactive in initiating socialization with colleagues due to cultural values, however future researchers may test this model in diverse contexts to rigorously understand the relevance of this model across the globe.

### Limitations & Methodological Suggestions

Some of the methodological limitations along with ideas for future suggestions are summarized in this section. First, while data for this study were collected from a large number of respondents, however a cross-sectional design was integrated, which might have led to little deviation in results. Future researchers are suggested to integrate longitudinal research designs for objectively measuring the impact of excessive work time on other outcomes. Moreover, data were collected from a single source i.e. employees. While employees can provide some understanding about their change experiences, it might be advisable for future researchers to use two-source data, and understand entrepreneurial behavior from supervisors’ perspective as these individuals can provide a holistic and unbiased perspective about changes. Third, data for this research has been collected from employees working at various levels within organizations for a holistic understanding of employee changes, however group-level differences were not compared during the analysis based on objectives of this research. Future researchers may enhance understanding about these relationships by focusingg on key differences between employees of variousage groupss or those working at different managerial level positions.

### Practical Implications

Using the findings of this research, practical implications are offered in this section. First, organizations have initiated new and rigorous processes for ensuring the timely completion of their goals after the Covid period. While these processes focus on the performance-based perspective, it has led to additional challenges for employees as they are required to spend a lot of time on these goals beyond their office hours, which is likely to lead to a detrimental impact on the health and mindset of employees in the long run. Hence, organizations are recommended to remain vigilant about this aspect and ensure maintaining specific hours for work purposes only. In this regard, HR departments may refine policies and explicitly share it across the organizations for ensuring the implementation of this aspect. Second, excessive work time is likely to prohibit employees from engaging in informal socialization at workplaces. While too much informal socialization is not suitable for organizations, it is important to maintain the right balance where employees get some time and opportunity regarding having informal discussions with each other as such discussions help them in understanding each other in a better way. In view of the same, organizations are recommended to provide some opportunities for informal interactions such as they may organize arrange occasional dinners for them. Alternatively, they may dedicate specific time (e.g. 1 hour) where employees can have lunch and gatherings with each other. Third, organizations need to think about improving resilience among employees, especially after the Covid period when things and ways of work have drastically changed as resilience facilitates employees in dealing with uncertain situations. For this, HR departments may organize regular training programs for employees to facilitate them in developing new skills and competencies which can help them in continuous learning and adaptation to situations.

## Conclusion

Using the social identity theory, this research has investigated the outcomes of excessive work time on the entrepreneurial behavior of employees. Moreover, it further investigated the mediating role of formal socialization and creative self-efficacy and the moderating role of resilience in this relationship. Based on the analysis of empirical data using the SEM, empirical findings provide an adequate understanding of the direct negative linkage between excessive work time and the entrepreneurial behavior of employees. Also, the statistical results indicated significant intermediate role of formal socialization and creative self-efficacy in this relationship, thus suggesting an essential role of interpersonal interactions. Overall, the findings of this research provide a basis for understanding how excessive work time and focus may lead to the development of entrepreneurial behavior in employees. Also, it helps in understanding the positive aspect of formal socialization patterns, which may be used for further developing theory in the future.

## Data Availability

The datasets generated during and/or analyzed during the current study are available from the corresponding author on reasonable request (Mumtaz & Aqif, 2023).
